# Detection of persistent low IgG avidity–an interpretative problem in the diagnosis of acute toxoplasmosis

**DOI:** 10.1371/journal.pone.0284499

**Published:** 2023-04-13

**Authors:** Petr Kodym, Zuzana Kurzová, Dagmar Berenová, Marek Malý

**Affiliations:** 1 National Reference Laboratory for Toxoplasmosis, National Institute of Public Health, Prague, Czech Republic; 2 National Institute of Public Health, Unit of Biostatistics, Prague, Czech Republic; Qatar University, QATAR

## Abstract

**Objectives:**

For the proper diagnosis of toxoplasmosis it is essential to determine the stage of the infection, for which the most preferred method is IgG avidity test. The avidity index (AI) should initially be low (AI≤0.3) in the acute phase and increase during the infection. However, persistent low avidity can occur in patients with latent toxoplasmosis, which can complicate the interpretation of the results. The aim of the study is to explain the causes of this phenomenon.

**Methodology:**

A retrospective study was carried out with 717 serum samples collected from 442 patients from the categories of pregnant and non-pregnant women, men, and newborns + infants (age < 0.5 year). The trends of AI kinetics were evaluated in repeatedly examined patients. The frequency of cases with low avidity was compared in individual categories of patients and in groups of people with acute and non-acute toxoplasmosis.

**Results:**

The proportion of patients with initially low avidity was 42.1% in the acute toxoplasmosis group while it was 13.0% in the non-acute groups. In uninfected newborns with anti-*Toxoplasma* antibodies transmitted from the mother, a decrease in IgG avidity levels over time was observed, resulting in 29.2% of samples showing low (improper) avidity. While the dynamics of IgG avidity and the frequency of cases of improperly low avidity were similar in men and pregnant and non-pregnant women, the category of newborns and infants differed substantially for these indicators.

**Conclusions:**

Due to acceptable specificity and negative predictive value, high avidity can rule out acute toxoplasmosis, but moderate sensitivity complicates the possibility of its confirmation. The results of the avidity test must be interpreted in the context of the results of other methods.

## Introduction

Toxoplasmosis is a globally widespread parasitosis caused by the protist *Toxoplasma gondii* (Apicomplexa), with a characteristic course of infection in two clinically distinct phases: acute and latent. Toxoplasmosis is most often asymptomatic in immunocompetent subjects, but more or less severe clinical signs such as lymphadenopathy, low-grade fever and malaise that may occur are tied to the acute phase. Possibly the most serious consequence of toxoplasmosis is that in pregnant women the acute phase of infection can result in transmission to the foetus. In contrast, the later latent (sometimes termed as chronic) phase runs in mothers and other patients a course free of any specific clinical signs, and transmission of the infection to the foetus in pregnancy does not occur. Due to permanent antigen stimulation, persons with toxoplasmosis usually keep lifelong specific antibodies [1–3]. Diagnosis must therefore rely primarily on serological tests, while the use of molecular genetic methods is limited by tissue localisation of the toxoplasma and only by a short period of parasitaemia. A key moment in toxoplasmosis diagnosis is determination of the phase of infection. Acute and latent toxoplasmosis are usually distinguished on the basis of the kinetics of anti-*Toxoplasma* IgM, IgA and IgE, the presence of which is considered a marker of the acute phase. However, this principle is limited by fairly frequent cases of overlong persistence of these isotypes, often longer than the duration of the acute phase [4]. Hedman [5] presented a method for differentiating between acute and latent phases of infection, based on measurement of antigen binding avidity. This is described as the aggregate strength by which a mixture of polyclonal IgG molecules reacts with multiple epitopes of the proteins. Functional binding affinity of anti-*Toxoplasma gondii* (*T*. *gondii*) IgG increases progressively after immunity from infection and is otherwise referred to as maturation of the humoral immune response [6–8]. Currently, IgG avidity is the most useful auxiliary or confirmatory test in IgM positive cases, determining with some degree of certainty, in cases of high avidity, that the first infection was ongoing for at least 3–4 months. One of the most useful utilities for measuring IgG avidity is based on the hypothesis that high-avidity results, especially in case of first examination of pregnant women performed at the beginning of pregnancy, rule out the risk of foetal transmission during pregnancy [9,10]. Nevertheless, it transpires that even IgG avidity testing has its pitfalls. Maturation of the humoral immune response can be delayed; according to [11], 26.4% of patients displayed a low avidity index even 1 year after infection. This raises the question if, in some patients, a lack of maturation of avidity could last forever [11]. A number of factors including marked heterogeneity of antibody responses, gestational age and antiparasitic treatment among others may impact the production *T*. *gondii*-specific antibodies and IgG avidity maturation [12]. As a rule, IgG avidity is taken very seriously in diagnostic practice; if the values are low, especially in pregnant women, the resulting alarm among non-specialist doctors can often be highly stressful for the patient even when other tests do not indicate acute toxoplasmosis. However, if the results of other tests are inconclusive, recognising "false" low avidity can be quite problematic.

The objectives of this study are to find out what causes can lead to the fact that the avidity index values remain low for a long time after infection and the avidity test provides incorrect results regarding the phase of infection. It was needed for this purpose to ascertain the course of humoral response, and the frequency of its delay in not only pregnant women but also the category of persons where long-term monitoring of the kinetics of avidity indices has no diagnostic value, and where there is a paucity of data in the literature. The study also comprises newborns and infants up to six months of age, non-pregnant women and men in both acute and latent phases of the disease. A better understanding of the course of antibody response is of importance for correct interpretation of serology.

## Materials and methods

### Study design

A retrospective study was carried out at the National Reference Laboratory for Toxoplasmosis, Prague (NRL TOXO) using serum samples which were collected in various medical facilities from patients suspected for toxoplasmosis and submitted to NRL TOXO for examination. Serological and clinical anonymised data were retrospectively extracted from patient databases, samples eligible for the study were selected and, if necessary, missing serological examination (IgG avidity) was completed. The study was conducted in accordance with the Declaration of Helsinki and the national legislation. The protocol was approved by the ethical committee of the National Institute of Public Health (Project identification code: SZU/12443/2021).

Clinical and laboratory data were collected at the baseline examination. A total of 717 serum samples collected in the 4-year period between November 5, 2015 and November 5, 2019. A single sample was available from 299 patients, 143 patients were tested repeatedly (2x: 82 patients, 3x: 30; 4x: 15, 5x: 4; 6x: 7; 7x: 3; 9x: 1; 12x: 1); their median follow-up period was 0.24 years, with a minimum of 0.06, and a maximum of 3.8 years.

### Characteristics of the patients

A total of 442 patients were included. The following diagnoses and reasons for examination were indicated: follow-up due to clinical toxoplasmosis (39.1% of samples), screening in pregnancy (18.7%), differential diagnostics in patients with other diagnoses (12.8%), screening after birth (11.1%), lymphadenopathy and other unspecific symptoms (6.1%) and others. The selection of samples is due to the structure of the material sent to the reference laboratory with an increased proportion of atypical and problematic samples.

The study group consisted of four categories, reflecting the purpose of examination as well as age (in years) and gender structure of patients investigated in NRL TOXO:

1. Non-pregnant women (*N* = 184, age: range 5–80, median 34.2);
2. Pregnant women (*N* = 141, age: range 19–44, median 31.5);
3. Newborns and infants up to six months of age (*N* = 82, 43 boys/39 girls; age: range 0.00–0.19, median 0.01). Three of these subjects, two girls and one boy, were congenitally infected; the others were *Toxoplasma*-free;
4. Men (*N* = 35, age: range 6–62, median 33.4).

The characteristics at the time of taking the first sample (age, pregnancy) were decisive for the classification of individuals into categories.

### Serological tests

Complement-fixation test (CFT) was performed with ingredients supplied by TestLine Clinical Diagnostics Ltd., Brno, Czech Republic: antigen (TOXO-CF-Ag lyophil) produced by the tween-ether preparation [13] from cultivated tachyzoites, amboceptor–rabbit serum containing antibodies against sheep erythrocytes (CF–AMBOCEPTORset), guinea-pig complement (CF–COMPLEMENT) and barbital buffer (Barbital buffer for CF– 5× conc.) according to the manufacturer-recommended standard procedure [14,15]. The samples are titrated, the last dilution of serum providing the positive reaction is declared as the resulting CFT titre. While titre 1:4 means an equivocal result, titres of 1:8 and higher are considered positive.

ELISA tests for anti-*Toxoplasma* IgG (EIA Toxoplasma IgG), IgM (EIA Toxoplasma IgM) and IgA (EIA Toxoplasma IgA) provided by TestLine Clinical Diagnostics Ltd., Brno, Czech Republic were used for detection of class-specific antibodies. All serological techniques are CE-IVD marked. In contrast to “sandwich-type” IgG ELISA, IgM and IgA tests are based on “capture” technique. The final phases of both types of procedures include incubation with chromogen–substrate solution containing tetramethylbenzidine and hydrogen peroxide as well as absorbance measurement with ELISA-reader at 450 nm wavelength.

For evaluation of test results of IgA and IgM ELISA tests, positivity index (PI) is calculated as a ratio of absorbances of tested sample and of cut-off control. Positivity index higher than 1.1 indicates positive results, samples with PI lower than 0.9 are considered negative, values in the interval of [0.9;1.1] lie in “grey zone” of equivocal results.

Semiquantitative results of IgG ELISA were expressed in IgG international units (IU). Absorbance of 4 calibrators, which are provided as a component of diagnostic kits (negative control: 0.1 IU/mL, cut-off: 6 IU/mL, positive control: 60 IU/mL, calibrator 4: 240 IU/mL), were measured and the resulting values served as data for the calibration curve, by which Magellan™ software (Magellan 5 version, Tecan Austria GmbH, Grödig, Austria) of the Sunrise™ Tecan ELISA reader converted the absorbance values of tested samples to international units. Samples with IgG levels higher than 240 IU/mL were diluted 1:10 and retested. The immunoenzymatic assay EIA *Toxoplasma* IgG (TestLine Clinical Diagnostics Ltd., Brno, Czech Republic) was also used, according to the manufacturer’s instructions, to determine the avidity of IgG. Briefly, the test was carried out on two parallels–diluted sera were dropped on plates A and B in an identical manner after the washing step and following incubation with sera. At that moment, an additional step was inserted: While plate A was subsequently incubated with Avidity solution 1 (stabilised urea–a component of the kit) at room temperature, plate B remained filled with washing solution for this period. The following procedure was again identical with both plates, as described in the IgG test. The resulting avidity index (AI) was calculated as a ratio of IgG concentration of each sera in IU/mL on plate A and plate B. Evaluation: AI ≤ 0.3 indicated low avidity and was considered as a marker of acute infection. AI values higher than 0.3 were interpreted as high avidity. Optimum IgG concentration for this test is 60 IU/ml in plate B. Sera with higher antibody levels should be diluted after the primary IgG semiquantitative test to adjust the IgG concentration to optimum value. According to instructions, in samples with IgG concentrations lower than 25 IU/ml, invalid results with improperly low avidity values can occur. Comparative Western blot (WB) IgG (LDBIO-Diagnostics, Lyon, France) was used to compare the antibody profile of mother and child. The difference in the child’s antibody profile compared to the mother’s profile means the production of the child’s own anti-*Toxoplasma* antibodies and thus the congenital infection of the child.

### Classification of the patients and their samples

Patients were primarily divided by age into neonates and adults. According to the first sample, they were classified based on CFT titer and IgG, IgM and IgA test results. IgG avidity was not considered in the classification. Furthermore, data on possible clinical symptoms were assessed. The kinetics of antitoxoplasmic antibodies was monitored in repeatedly examined patients. According to all the above data, the patients were classified retrospectively into 4 groups representing various forms and stages of *Toxoplasma* infection.

The newborns and infants (age at first sample collection < 0.5 year) are divided into two groups:

- **Newborns with congenital toxoplasmosis (NA)** (*N* = 3, 1 boy/2 girls, age: range 0.00–0.04, median 0.00), the diagnosis was established by a doctor—an infectious disease specialist. Two neonates were asymptomatic, infection was detected through maternal seropositivity. The third was diagnosed with a congenital malformation of the central nervous system. The diagnosis was confirmed in all of them by comparative WB IgG and in one by positivity of anti-toxoplasmic IgM and in one by IgA. Until the end of the follow-up (3, 10, and 47 months for individual newborns), a definite positivity of IgG persisted in all of them.
- **Seropositive uninfected newborns and infants (NU)** (*N* = 79, 42 boys/37 girls, age: range 0.00–0.19, median 0.01), results of serological tests indicate the transmission of maternal antibodies, negative IgM, IgA, decrease of antibody levels in subjects followed for a sufficiently long time until seronegativity. The longest detected persistence of antibodies was up to the age of 7 months.

Patients representing postnatal toxoplasmosis are classified into two groups:

- **The recent acute toxoplasmosis group (recent)** (*N* = 76, 7 males/69 females, age: range 9–68, median 31.4) with significantly positive CFT, IgG, IgM and IgA. Recent acute toxoplasmosis was also confirmed by one or more of the following criteria: an increase in the level of anti-toxoplasmic antibodies of the class IgG (24x), IgM (15x), IgA (6x), rise of CFT titers (13x) and clinical symptoms—lymphadenopathy (11x) and primary infection during pregnancy leading to the birth of an infected child (1x). Of the remaining 22 patients, included only for strong serological results (medium CFT titers—1:128–1:256, medium IgG anti-*Toxoplasma* values: 100–250 IU/ml, significantly positive IgM and IgA—PI≤2) 19 have only one examination.
- **The past (latent) toxoplasmosis group (PAST)** (*N* = 284, 28 males/256 females, age: range 5–80, median 32.7) with negative IgM and IgA and stable or declining IgG or CFT titers. The group consists of 3 subgroups:
  **LL**: latent toxoplasmosis (*N* = 133, 12 males/121 females, age: range 9–80, median 34.2) with low or equivocal (stable or declining) levels of anamnestic antibodies: low complement-fixation test (CFT) titres, IgG test equivocal or positive, both IgM and IgA tests negative;
  **LH**: latent toxoplasmosis with medium or high levels of CFT titres (*N* = 50, 8 males/42 females, age: range 9–78, median 32.5), IgG test equivocal or positive, both IgM and IgA tests negative, both IgG levels and CFT titers stable or declining;
  **PR**: post-recent toxoplasmosis (*N* = 101, 8 males/93 females, age: range 5–63, median 30.9) with equivocal or positive (stable or declining) CFT, IgG and IgM tests and with negative IgA test; patients show no clinical signs.

Any second or subsequent sample from each patient was evaluated separately according to the above criteria and the course of the infection and was not necessarily classified in the same category as their previous sample.

Seronegative samples were not included. The representation of individual groups and their serological parameters is shown in the Table 1.

**Table 1:** Serological parameters of 717 samples categorised in six groups and subgroups according to the toxoplasmosis status.

Group		RECENT	PAST			NA	NU
Subgroup		N = 158	LL N = 145	LH N = 75	PR N = 186	N = 23	N = 130
Parameter							
**CFT** **(titre)**	Minimum	1:32	1:4	1:64	1:8	1:16	1:4
	Maximum	1:4096	1:32	1:1024	1:1024	1:4096	1:1024
**IgG** **(IU/ml)**	Median	204	43	107	94	102.0	61.7
	Minimum	36	6	11	6	19.2	6
	Maximum	>3 000	229	>3 000	>3 000	>3 000	>3 000
**IgM** **(PI)**	Median	2.95	0.33	0.56	1.60	0.56	0.07
	Minimum	0.96	0.05	0.07	0.91	0.05	0.01
	Maximum	9.43	0.89	0.89	6.06	1.35	0.59
**IgA** **(PI)**	Median	1.82	0.15	0.26	0.40	0.24	0.12
	Minimum	0.92	0.03	0.04	0.13	0.09	0.02
	Maximum	7.22	0.84	0.86	0.88	3.32	0.76
**IgG avidity** **(AI)**	Median	0.36	0.62	0.62	0.51	0.35	0.47
	Minimum	0.04	0.11	0.01	0.03	0.08	0.05
	Maximum	0.94	0.95	0.98	0.96	0.85	0.96
	low avidity [% of samples]	41.8	6.2	13.3	25.8	43.5	29.2

Group RECENT—recent acute toxoplasmosis; Group PAST—past (latent) toxoplasmosis; subgroups: LL—latent toxoplasmosis with low CFT titre, LH—latent toxoplasmosis with high CFT titre, PR—post-recent toxoplasmosis; Group NA—newborns with congenital toxoplasmosis; Group NU—seropositive uninfected newborns; CFT—complement-fixation test, PI—positivity index, AI—avidity index.

### Data analysis

Continuous data are characterised by medians, and categorical data by percentages. Statistical analysis of repeated avidity measurements was performed using a random-effects linear model with robust estimator of variance. The model yielded estimates of regression parameters, including slopes of resulting regression lines, and, with the aid of appropriate predictors, enabled testing of between-groups and between-categories differences. Mann-Whitney test was used for determining if there are differences in the central tendency of two groups. The statistical significance level was set to 0.05. Statistical software Stata 14.2 (StataCorp LP, College Station, TX, U.S.A.) was used for data management and analysis.

## Results

### Low IgG avidity in different groups and categories

Samples showing low avidity of anti-*Toxoplasma* IgG are present in all groups representing various phases of infection (see Table 1), but their percentage differs significantly. In the RECENT group (158 samples), 41.8% of samples showed a low avidity, compared to 16.5% in the PAST group (406 samples). The percentage of samples with low avidity in the PAST group decreases with time since infection. It is highest in the PA subgroup and lowest in the LL subgroup, comprising samples in which antibody levels have already significantly decreased. The differences between the three subgroups PR, LH, and LL are statistically significant (p = 0.002). When evaluated according to patients on the basis of their first sample, low avidity was found in 42.1% of patients in the RECENT group, compared to 13.0% in the PAST group. In the NA group of congenital toxoplasmosis the proportion of low avidity samples was 43.5%. It is worth noting that in the NU group of seropositive uninfected newborns and infants this figure was as high as 29.2%.

The median of avidity index is significantly lower in the RECENT group (0.36) than in the PAST group (0.58), *p*<0.001 (Mann-Whitney test). However, the extreme values are practically not different: the minimum and maximum AI values in the RECENT group were 0.04 and 0.94, while in the PAST they were 0.01 and 0.98, respectively.

As Table 2 shows, based on the first available sample, the proportion of low IgG avidity does not differ significantly among categories of non-pregnant women, pregnant women, and men in either the RECENT group (*p* = 0.984) or the PAST group (*p* = 0.733). There are no differences between non-pregnant and pregnant women in either of the two comparisons. All 3 newborns with congenital toxoplasmosis had low initial IgG avidity. Among 79 seropositive uninfected newborns, the proportion of low IgG avidity results was 19.0%, higher than in the PAST group.

**Table 2:** Percentage of low IgG avidity results in the first sample in three categories of patients with recent acute toxoplasmosis and with past toxoplasmosis.

Group	RECENT		PAST	
Category	Low Avidity	Total (N)	Low Avidity	Total (N)
Pregnant women	11 (40.7%)	27	16 (14.0%)	114
Non-pregnant women	18 (42.9%)	42	19 (13.4%)	142
Men	3 (42.9%)	7	2 (7.1%)	28
TOTAL	32 (42.1%)	76	37 (13.0%)	284

Group RECENT—recent acute toxoplasmosis; Group PAST—past (latent) toxoplasmosis.

### Relationship between IgG avidity and antibody concentration

In Table 3, the percentage of samples showing low IgG avidity, in groups representing recent acute and past toxoplasmosis, and seropositive uninfected newborns and infants, is related to IgG concentration in samples. In the RECENT group, there is no statistically significant difference among IgG concentration categories (*p* = 0.392) although category 50–99 IU/mL deviates to some extent. In the PAST group, the IgG concentration categories differ significantly (*p* = 0.002). A higher percentage of low IgG avidity is seen in the groups of samples with highest IgG values (100–249, 250 and more IU/mL) in which the shortest time from *Toxoplasma* infection to sample collection is expected. Categories with moderate IgG concentrations (25–49 and especially 50–99 IU/mL) show lowest percentage of low avidity samples. The very highest percentage of low IgG avidity samples is found in the category with the lowest IgG content, which does not meet the limits of the avidity test. No significant association between IgG concentrations and proportion of samples with low IgG avidity is seen in seropositive uninfected newborns and infants (*p* = 0.933).

**Table 3:** Percentage of low anti-*Toxoplasma* IgG avidity in 694 samples categorised by IgG concentration.

Group	RECENT			PAST			NU		
IgG (IU/mL)	Low Avidity		Total (N)	Low Avidity		Total (N)	Low Avidity		Total (N)
	[N]	[%]		[N]	[%]		[N]	[%]	
**6–24**	-	-	0	16	29.6	54	9	31.0	29
**25–49**	3	50.0	6	12	14.1	85	8	33.3	24
**50–99**	7	26.9	26	9	7.4	122	8	23.5	34
**100–249**	30	44.8	67	25	21.6	116	10	30.3	33
**250+**	26	44.1	59	5	17.2	29	3	30.0	10
**TOTAL**	66	41.8	158	67	16.5	406	38	29.3	130

Group RECENT—recent acute toxoplasmosis; Group PAST—past (latent) toxoplasmosis; Group NU—seropositive uninfected newborns.

### Sensitivity, specificity, negative and positive predictive values of IgG avidity test

There is only partial agreement between the results of the IgM and IgA ELISA and the IgG avidity test as potential markers of acute toxoplasmosis. In samples with positive IgM and IgA, low avidity was obtained in 33.1% and 41.8%, respectively, while with negative IgM and IgA, high avidity was obtained in 83.7% and 80.4%, respectively.

Working on the assumption that the IgG avidity should be low in all samples of the RECENT group comprising samples of recent acute toxoplasmosis, while it must be high in the PAST group, raw diagnostic parameters of the IgG avidity test were calculated (Table 4). Unlike the common practice, no other diagnostic methods were taken into account. When evaluating on the basis of the patient, a slightly higher specificity– 87.0% (95% CI: 82.5%–90.7%) and negative predictive value– 84.9% (95% CI: 80.2%–88.8%) were achieved, although the remaining parameters do not differ substantially.

**Table 4:** Diagnostic parameters of low IgG avidity (regardless of other tests) upon confirmation or exclusion of acute toxoplasmosis.

Parameter	Value	95% Confidence Interval
Sensitivity	41.8%	34.0%–49.9%
Specificity	83.5%	79.5%–87.0%
Positive predictive value	49.6%	40.8%–58.4%
Negative predictive value	78.7%	74.5%–82.4%

### Trends in kinetics of the avidity index in course of toxoplasmosis

**Fig 1:**
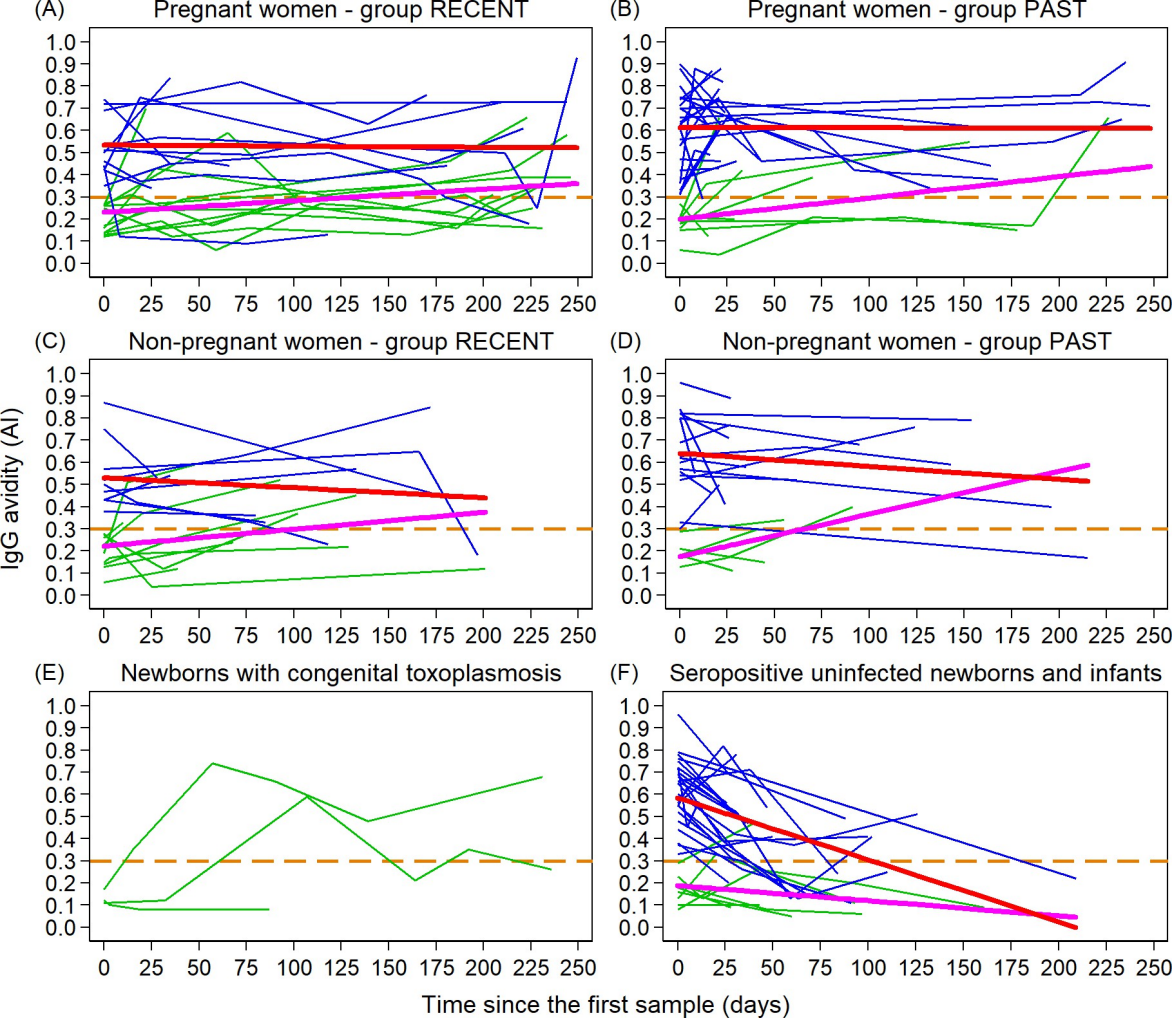
Kinetics of the avidity index (AI) in individual patients followed-up with 2–6 samples, starting with low (AIF0A30.3—green) or high (AI>0.3—blue) IgG avidity in the course of toxoplasmosis. The trend lines obtained from the regression model are marked in purple for patients with low avidity, and in red for patients with initial high avidity. Graphs (A) and (B) represent pregnant women, graphs (C) and (D) non-pregnant women, while graph (E) includes congenitally infected newborns and (F) seropositive uninfected newborns and infants up to six months of age. Graphs (A) and (C) are related to the recent acute toxoplasmosis, graphs (B) and (D) to the past toxoplasmosis. The low avidity threshold of 0.3 is indicated by the dashed orange line.

In the initial phase of the infection, avidity of anti-*Toxoplasma* IgG shows an evident trend to increase from the original low values. The increase of the avidity index was faster or slower in individual patients. The rising phase had a duration of 2–8 months, whereas the high avidity cut-off was exceeded mostly after 1–4 months (median: 57 days). In the patients with initially low avidity, the growth of IgG avidity was corroborated by the positive values of regression line slope. Increasing trend proved to be statistically significant in pregnant women from the RECENT group (Fig 1(A), *p* = 0.042) and in both pregnant (Fig 1(D), *p* = 0.049) and non-pregnant (Fig 1(D), *p* = 0.016) women from the PAST group, and insignificant (*p* = 0.236) in non-pregnant women from the RECENT group (Fig 1(C)).

After reaching the maximum values of the avidity index, the initial trend of increase switches into the trend of stability or a slow decline, but only exceptionally the AI falls into “low avidity” range. This scheme does not differ much between the follow-up of patients of the RECENT group (Fig 1(A) and 1(C)) and the PAST group (Fig 1(B) and 1(D)), or between the categories of non-pregnant women, pregnant women and men. The trend of decrease of AI in patients starting with high avidity was expressed by negative slope. The decrease is statistically significant in non-pregnant women from the PAST group (Fig 1(D), *p* = 0.016) and statistically not significant in the three remaining groups of women (Fig 1(A), 1(B) and 1(C)). In the PAST group, there were statistically significant differences in the trend between regression lines for women with initial low and high avidity, both in pregnant and non-pregnant (Fig 1(B) and 1(D)) women (*p* = 0.025, and 0.005, respectively). In the RECENT group, some differences can also be seen, but they are not statistically significant. Significant differences were not detected in mutual trends between groups RECENT and PAST or between pregnant and non-pregnant women. The trends do not appear to be different in men although the number of repeatedly tested patients is not sufficient for graphical processing in this category.

A specific situation is seen in the category of uninfected newborns whose anti-*Toxoplasma* antibodies are transmitted from their mothers. Here, the general trend during the whole period of follow-up is decrease of AI, not infrequently converting from “high” to “low” avidity (Fig 1(F)). The decrease is statistically significant for both patients with low and high initial AI values (*p* = 0.001, *p* < 0.001) and is significantly sharper in the latter group (*p* < 0.001).

Three newborns were congenitally infected and synthesised anti-*Toxoplasma* antibodies which were characterised with very low initial IgG avidity (Fig 1(E)).

## Discussion

As the anti-*Toxoplasma* IgG avidity test is crucial in determining the stage of infection and thus the risk of foetal harm, the course of avidity is most closely monitored in pregnant women. If the IgG avidity is high at the first examination, it is clear that this is not a recent acute infection and no further monitoring is performed. It also makes no sense to monitor IgG avidity in other patient categories and to perform repeated examinations of non-pregnant women, men and children. Therefore, information about the dynamics of the avidity index for these groups is missing.

It is well known that the initial phase of the infection is characterised by a rise in IgG avidity caused by maturation of antibody immunity [6]. Extremely low avidity (AI < 0.1) can be indicative of very recent infection. According to [16], mean IgG avidity increased from 0.035 in the first month to 0.387 one year after onset of infection.

The bigger surprise was that on reaching its zenith, there follows a phase of persistent values or a decline. As IgG levels decrease, avidity test results tend to have lower AI values, but they rarely fall into the “low avidity” range, generally associated with the onset of infection. This process of rise and fall in IgG avidity during the course of toxoplasmosis was observed in both the pregnant and non-pregnant women categories; the regression line slopes of individual categories had no statistically significant differences. It is known that if avidity is low at the first collection, it may not necessarily rise immediately during subsequent ones. In the Norwegian study, 43% of pregnant women had stable low avidity during the observation period [17]. Because women with initial high avidity were not included in the study, the decline is only slightly indicated in one of the graphsA wholly different trend was detected amongst the uninfected newborns who have transmitted maternal anti-*Toxoplasma* antibodies, so that their IgG levels reflect the maternal values [18]. The level of transferred IgG is gradually reduced by their degradation. Antibodies persisted until 7 months of age. Subsequent conversion to seronegativity is evidence to rule out toxoplasma infection, but samples collected after it could not be included in the IgG avidity study. Because the maturation of the antibody response does not occur in them, no characteristic increase in AI can be observed in neonates with initial low avidity, whereas individuals with high avidity show a decrease. Therefore, the overall proportion of the resulting low avidities reaches 29.2% in the present study. The low avidity of anti-*Toxoplasma* IgG does not preclude transferred maternal antibodies, so monitoring IgG avidity in newborns makes no sense in diagnosing congenital toxoplasmosis [see 19]. Studies that argue the opposite [20,21] paid little attention to the dynamics of the avidity index of transmitted antibodies. In contrast, congenitally infected neonates are characterised by an initial low IgG avidity with a subsequent long-term upward trend. Congenital toxoplasmosis was more frequent in newborns with low (94.1%, 16/17) than with high (51.6%, 16/31) avidity [21]. This study suggests that the speed of antibody response maturation does not depend on the patient categories such as non-pregnant and pregnant women or men which have no significant differences in the proportion of low IgG avidity. There is, nonetheless, controversy concerning data on the significance of treatment. Whereas some authors state that treatment retards maturation and decreases mean avidity index values [16,22,23], other studies have not confirmed this effect [9,12,17]. A delay in maturation of Toxoplasma-specific avidity until the late phase of pregnancy has been described [23]. The determination of IgG avidity is a highly useful means towards identifying the infection timeline. A great advantage of the test is that high avidity can be used to exclude recent acute infection; its disadvantage is that low avidity often persists for too long and thus cannot reliably verify an acute infection [17].

The biggest problem is that there are no clinical or serological definitions of acute and chronic infection [11]. Even a rise in the levels or titers of anti-toxoplasmic antibodies is not a guaranteed reliable sign of the acute phase. The dynamics and time of rise or fall of individual markers is highly variable and differs from patient to patient, and therefore cannot be as robust and reliable confirmation of recent acute infection as the gold standard–seroconversion. Since no seroconversion was recorded in any of the patients in our series, the exact time since infection is not available. Therefore, cases of recent acute toxoplasmosis were selected according to other, less reliable criteria, but misclassification can occasionally occur. This shortcoming, which may have affected the results, is a limitation of the study.

It is not clear how long the acute phase lasts. Time could conceivably be measured by the duration of lymphadenopathy, although this is widely variable. Ho-Yen [24] monitored 185 patients: the vast majority (111; 60%) recovered within 2 months, and of these most made a recovery in 1 month. However, a quarter of patients (48) took 2–4 months to return to normal health and 15 (8%) took 4–6 months. A substantial proportion of patients (11; 6%) do not take much longer to recover. If we use the period during which foetal infection is possible as a baseline, then the acute phase, associated with parasitaemia, would probably be considerably shorter. Innumerable data suggest that the placenta acts as a source of parasites which are transmitted to the foetus almost immediately after maternal infection or with a delay of several weeks or longer [25,26]. Čermáková [27] concluded in accordance with literature sources that in routine examination the detection of protozoan DNA in blood samples is not possible more than 4 weeks following manifestation of the infection. During the parasitaemic period IgG avidity index seems to be extremely low: avidity reached a value of 7.2% in a single patient, who did not have a negative PCR test [27]. Ninety-seven percent of exploitable sera with very low IgG avidity indices ≤ 0.05 (Liaison XL, low avidity threshold: AI≤0.3) actually corresponded to recent infection [28].

A further problem lies in the lack of complete agreement between the determination of acute toxoplasmosis by the two methods: low IgG avidity on the one hand, and levels of antibodies of different classes on the other. Although isotype switching [29] and IgG avidity maturation [6] are parallel processes of antibody response, there is no causative connection between the two; this is why there is only partial correlation between low avidity of anti-*Toxoplasma* IgG and positivity of IgM, IgA and IgE antibodies. Furthermore, there are marked individual differences. IgM, IgA and IgE antibodies are as a rule detectable for a longer period than low IgG avidity [30] which can result in a high avidity reading from some samples of the RECENT group. There are cases, by no means unique, when the increase of IgG avidity is slower than the decline in antibody levels and avidity remains low even in the event of latent or post-recent toxoplasmosis. Lefevre-Pettazzoni [23] found 0–66% of low avidity results from chronic infections quoted in 11 published studies. Only 62.5% of IgM-positive women had low-avidity IgG antibodies [31]. Among pregnant women with positive IgG and IgM only 7.1% had low avidity [32]. On the other hand, 20% of serum samples with low avidity had negative results in the IgM ELISA test; recently acquired infection was not, however, confirmed in any of these cases [33].

A major problem when interpreting results is that due to the relatively frequent long persistence of low IgG avidity, the test is not always indicative of the time since infection [34]. The cut-off value of high IgG avidity is usually reached at month 4 after onset of infection [16] but low IgG avidity can persist for seven or more [17] or nine [33] months after infection, and sometimes one year [35], two years [36] or even 11 years [11].

According to [17], 42% of women had stable low IgG avidity during the observation period. In the present study, delayed maturation was discovered in 13.0% of patients with acquired non-acute toxoplasmosis, which approaches the 15.4% quoted by [37]. Low avidity, therefore, is seen not only in the category that represents acute and possibly post-recent toxoplasmosis but also in samples with high or low levels of unequivocally anamnestic antibodies. The number of persistent low avidity samples in a group depended on the time since onset of infection. This explains why the LL subgroup, which represents latent toxoplasmosis in persons presumed to have been infected years ago, has only 6.2% of low avidity samples, whereas in the PR subgroup, in which acute toxoplasmosis is transforming into the latent form, the proportion is 25.8%. The figures confirm what is shown on the graphs of long-term AI kinetics: these are not cases of persistent low IgG avidity but delayed maturation of antibody response which, perhaps even after a long period of time, reaches high avidity IgG antibody results.

This connection is also seen in relation to concentrations of anti-*Toxoplasma* IgG which have a declining trend over time from start of infection, resulting in a decline of low avidity samples as well. At very low levels of IgG antibodies which have not yet been completely degraded, for example in uninfected neonates, the percentage of low-avidity samples increases. It is possible that the technical issue also plays a role here: If the antibody level does not reach the manufacturer-prescribed values and test-validity criteria are not met, the reaction will be inadequate and avidity may be read as low. Results from such samples should be viewed as dubious. Conversely, in samples with high IgG content appropriate dilution of serum is suitable for testing of avidity [38].

All of these problems are associated with diagnostic parameters of standalone determination of anti-*Toxoplasma* IgG avidity. The results presented here show that especially sensitivity and positive predictive value evaluated mechanically (low avidity = recent acute toxoplasmosis) out of context with other diagnostic methods, are modest. The overall analyses of serological parameters, taken alone, are not applicable to laboratory diagnosis [10]. On the other hand, acute toxoplasmosis can be fairly reliably ruled out in patients with high IgG avidity, as shown by the reasonably good specificity and negative predictive value in the present study, even evaluated in this manner. In actual practice it makes sense to determine IgG avidity in selected samples only, and not, for example, in IgM negative ones. Avidity tests have proved highly satisfactory performance with careful selection of samples in reference panels on the basis of clinical and biological data, together with results of previous or subsequent sera tested by multiple IgG and IgM assays. For instance, comparison of Vidas (bioMérieux, Marcy l’Étoile, France), Architect (Abbott Laboratories, Wiesbaden, Germany) and Liaison® (DiaSorin, Saluggia, Italy) systems have revealed sensitivity 84.4–93.8%, specificity 99.3–100%, positive predictive value 95.2–100% and a negative predictive value of 97.8–99.8% [39]. In our study, all determinations of anti-Toxoplasma IgG avidity were performed using the EIA Toxoplasma IgG test, TestLine Clinical Diagnostics, and all results refer only to the parameters of this particular test. A limitation of the study is that the samples were not investigated by any other commercial test.

The utility of avidity as a marker of foetal infection was verified in comparison with PCR from amniotic fluid. Low IgG avidity test showed 77.8% sensitivity and 81.5% specificity for the prediction of the presence of *T*. *gondii* DNA in the amniotic fluid [40]. IgM-negative samples with high avidity were indicative of chronic infections with excellent specificity (97.6%) and negative predictive value (95.6%) [41], while the best assay combinations, measuring IgM and IgG avidity, reached diagnostic specificities of 99% and sensitivities of 95% or higher [42].

Barros [10] categorised the *T*. *gondii* infection into distinct phases, referred to as acute (1, 2 and 3 months), early (4, 6 and 8 months) and late convalescent (10 and 12 months). Whereas the acute subgroup presented a homogenous serological pattern characterised by positive IgM along with low IgG avidity in 91% (ELFA and FC) and late convalescent infection rendered an opposite profile comprising of negative IgM together with high IgG avidity in 97%, a transitional profile with 49% low avidity was observed for early convalescent *T*. *gondii* infection. No assay combination was able to distinguish between acute and convalescent infections [42].

Some manufacturers of diagnostic kits have adopted the careful approach of elevating the threshold value of low avidity from the usual AI values of 0.2–0.3 to 0.5–0.6 [8] resulting in increased sensitivity, but also decreased specificity and predictive value of a positive test. The increased frequency of low avidity results can be confusing in diagnostic practice; on the contrary, if the avidity index values are very low, the specificity of the test is high, making recent acute toxoplasmosis probable.

If test results show low avidity it is important to consider the possibility of both acute toxoplasmosis and delayed maturation of immune response. The avidity test should not be used on its own [5,43]. It is necessary to interpret results rigorously and to assess if they make sense in the light of the avidity assay’s parameters and the results of other tests. In the event that high avidity is detected then acute toxoplasmosis can very probably be ruled out [17].

## Conclusions

IgG avidity assay is a very useful tool for determining the stage of infection. The problem is that as a result of delayed IgG response maturation, improperly low avidity is seen on average in 13.0% of patients with past toxoplasmosis. The present study shows that this incorrect result can occur in the following situations in particular:

- In samples with a very low content of *Toxoplasma* IgG which does not conform to test criteria.
- In uninfected newborns with decreasing levels of anti-Toxoplasma antibodies transmitted from the mother.
- In women (pregnant and non-pregnant) infected a long time ago, who may be in a phase of declining IgG avidity following a period of initial rise; however, AI rarely falls below the low avidity threshold at this stage.

On the other hand, the frequency of cases of improperly low avidity does not depend on the category of patients examined. Comparison of the course of antibody response maturation in various categories of patients has shown that in pregnant and non-pregnant women, and apparently also in men, the kinetics of IgG avidity is more or less the same.

The low sensitivity and positive predictive value of a standalone avidity assay can be managed by evaluation in the context of all diagnostic tests, while acceptable specificity and negative predictive value enable exclusion of an acute toxoplasmosis diagnosis, if avidity is high.

## Supporting information

S1 Table Avidity indices of all subjects in the study with the listed groups according to the stage of infection and gender categories. (XLSX)
